# Quantitative Modeling of Cerenkov Light Production Efficiency from Medical Radionuclides

**DOI:** 10.1371/journal.pone.0031402

**Published:** 2012-02-20

**Authors:** Bradley J. Beattie, Daniel L. J. Thorek, Charles R. Schmidtlein, Keith S. Pentlow, John L. Humm, Andreas H. Hielscher

**Affiliations:** 1 Medical Physics, Memorial Sloan-Kettering Cancer Center, New York, New York, United States of America; 2 Radiology, Memorial Sloan-Kettering Cancer Center, New York, New York, United States of America; 3 Biomedical Engineering, Columbia University, New York, New York, United States of America; University of Texas, M.D. Anderson Cancer Center, United States of America

## Abstract

There has been recent and growing interest in applying Cerenkov radiation (CR) for biological applications. Knowledge of the production efficiency and other characteristics of the CR produced by various radionuclides would help in accessing the feasibility of proposed applications and guide the choice of radionuclides. To generate this information we developed models of CR production efficiency based on the Frank-Tamm equation and models of CR distribution based on Monte-Carlo simulations of photon and β particle transport. All models were validated against direct measurements using multiple radionuclides and then applied to a number of radionuclides commonly used in biomedical applications. We show that two radionuclides, Ac-225 and In-111, which have been reported to produce CR in water, do *not* in fact produce CR directly. We also propose a simple means of using this information to calibrate high sensitivity luminescence imaging systems and show evidence suggesting that this calibration may be more accurate than methods in routine current use.

## Introduction

Cerenkov radiation (CR), first described by Pavel Cerenkov nearly a century ago, is produced when a charged particle travels through a dielectric medium at a speed greater than the phase velocity of light in that medium (i.e. greater than the speed of light in a vacuum divided by the refractive index of the medium) [1], [2]. These conditions produce a photonic shockwave somewhat similar to the sonic shockwave (i.e. sonic boom) associated with supersonic bodies in air. Cerenkov photons have a broad frequency spectrum with intensity inversely proportional to the square of the photon's wavelength within and extending somewhat beyond the visible range.

Recent renewed interest in CR began following the demonstration of detectable amounts of light emanating from a radionuclide bearing live mouse [3], [4], suggesting the possibility of exploiting this phenomenon for medical research and possibly clinical purposes. In this context, a number of radionuclides have been tested for CR production (e.g. F-18, N-13, Cu-64, Zr-89, I-124, Lu-177, Y-90, I-131) [5], [6] including some radionuclides, In-111 and Ac-225, that one might not, upon initial consideration, expect to produce CR owing to their lack of a sufficiently high velocity charged particle emission. In-111 decays via electron capture and emits only γ-rays with significant abundance. Ac-225 is a virtually pure α emitter, but α's in water become superluminal only at energies well beyond those of Ac-225's emissions. Never-the-less, experiments designed to measure CR conducted by multiple groups have detected light emanating from both In-111 and Ac-225 [5], [6]. However, to-date, clear evidence demonstrating that the Cerenkov mechanism is the source of this light has been lacking.

Of the potential biomedical uses of CR, the most commonly cited application is as a low cost, high throughput alternative to PET imaging [3], [4], [7] referred to as Cerenkov Luminescence Imaging (CLI). Other proposed applications include: an alternative to bremsstrahlung for imaging pure β^−^ emitting radionuclides [3], [7]; a higher resolution autoradiography method for high energy β's [7]; intra-operative or endoscopic imaging of targeted structures in humans [6]; an excitation source for various fluorophores [8], [9], [10]; and most recently a renewed interest in using CR as a light source for photodynamic therapy [11], [12]. In each of these applications there are also disadvantages to using a Cerenkov derived signal (e.g. limited half-life, ionizing radiation, poor tissue penetration). As such, it is yet unclear whether any of these new applications of Cerenkov imaging will prove to be clearly superior to extant techniques and enjoy widespread use.

However, one concrete and clearly advantageous proposed use of CR is as a means of validating the results of luminescence tomography reconstruction algorithms [3]. Thus far, manuscripts have been published using both SPECT [13] and PET [14], [15] imaging as validated reference standards. In these papers, the comparison of the reconstructed luminescence to the nuclear imaging reference was limited to a simple difference between centroid locations. One of these papers [15] looked at the linearity between the two signal intensities but did not establish a relationship in absolute terms that spanned the in vitro and in vivo conditions.

The work to be presented here seeks to establish such a cross-calibration between the signals derived from CR and nuclear tomographic imaging modalities, thus allowing nuclear imaging to better serve as a means of validating luminescence tomography reconstruction algorithms. Since PET and (in some cases) SPECT are already quantitative in terms of absolute radioactivity, establishing a cross-calibration amounts to determining the quantity of CR produced per unit radioactivity under imaging conditions and then measuring the light in absolute terms.

We accomplish this task using a set of computer models of CR production and apply the models to predict and tabulate the efficiency of CR production for a number of medical radionuclides under a variety of conditions affecting said efficiency. We also look at the intrinsic resolution of the Cerenkov light produced by these radionuclides. Experiments involving a subset of these radionuclides will be used to validate our model results. Moreover, we evaluate the CR production capacity of the two radionuclides for which this capability has been questioned, namely In-111 and Ac-225.

Finally, we propose a simple system that uses CR as a low intensity light source able to calibrate luminescence imaging systems thus avoiding the expense of specially calibrated sources, integrating spheres and the like. We present data suggesting that this approach may be more accurate than calibrations currently performed by manufacturers, including with regard to the calibration of spectral filters.

## Materials and Methods

### Model overview

The radionuclides to be considered here decay primarily by α, β^+^, β^−^ and γ emissions. Neutrinos, conversion electrons, Auger electrons, characteristic x-rays, bremsstrahlung radiation, annihilation photons, δ-rays, e^+^/e^−^ pairs and secondary electrons are also produced.

The α particles emitted by radionuclides generally are not of sufficient energy to be superluminal when transiting through water, biological tissues or other non-periodic mediums of moderate refractive indices, and should not produce CR. Likewise, the secondary electrons produced by the transiting α's are not of sufficient velocity because each electron receives only a small fraction, a maximum of 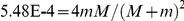, of the α's energy (where 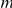 and 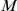 are the rest masses of the electron and α, respectively). Neutrinos, Auger electrons, characteristic x-rays and e^+^/e^−^ pairs (i.e. pair production), are all either not produced at significant quantities, are not of sufficient energy or do not interact with matter with sufficient efficiency to produce Cerenkov radiation. Bremsstrahlung radiation can extend into the visible spectrum and thus conceivably could be confused with Cerenkov radiation, but the amount within the visible range is expected to be negligibly small and its wavelength distribution would be dissimilar to the characteristic one over wavelength squared Cerenkov distribution and thus can be easily distinguished. This leaves β^+^, β^−^, δ-rays, conversion electrons and secondary electrons produced by both γ-rays and annihilation photons as the potential dominant sources of Cerenkov radiation. These are all, in essence, β particles (i.e. electrons or positrons) of varying origin. Over the range of β energies we are interested in here, there are negligible differences in the Cerenkov producing properties of β^+^ and β^−^ particles and no difference what-so-ever among β^−^, δ-rays, conversion electrons and secondary electrons [16].

Table 1 lists for each of the radionuclides to be modeled, the total abundance of each of the types of emissions at least some of which have sufficient energy to produce CR in water. Along with the half life and total abundance [17], we list the abundance of the portion of those emissions that are above the energy threshold of CR production in water (refractive index 1.33, threshold 263 keV [18]) and in mammalian tissue (refractive index of 1.4, threshold 219 keV [19]); these based on our own integrations of the β energy spectra [17]. For example, while the overall β^+^ abundance of F-18 is 97% the β^+^ with energy ≥263 keV is only 43% and ≥219 keV is 54%. Note that since annihilations photons have a kinetic energy of 511 keV, they are above the threshold for both refractive indices (1.33 and 1.4) and their abundance is twice that of the β^+^ total abundance.

**Table 1:** Total abundance and abundance of emissions having energy greater than CR thresholds in water and in biological tissues.

radio-nuclide	half life^a^	β^+^ (%)			β^−^ (%)			conversion electrons (%)			γ-rays (%)		
		total	1.33	1.4	total	1.33	1.4	total	1.33	1.4	total	1.33	1.4
C-11	20.4 m	100	69	77									
N-13	9.97 m	100	79	84									
O-15	122 s	100	90	93									
F-18	109 m	97	43	54									
Cu-64	12.7 h	18	9	11	39	11	15	<0.1			<0.1		
Ga-67	3.26 d							34	0	0	88	22	22
Ga-68	67.7 m	89	83	85				<0.1			4	4	4
Zr-89	3.27 d	23	17	19				<0.1			101	101	101
Y-90	2.67 d				100	89	91	<0.1			<0.1		
In-111	2.80 d							16	0	1	185	0	94
In-114m	49.5 d							81	0	0	22	6	6
In-114	71.9 s				100	85	89	<0.1			<0.1		
I-124	4.18 d	24	22	23				<0.1			99	99	99
I-131	8.03 d				100	36	35	6	2	2	101	98	98
Ac-225	10.0 d							67	0	0	7	0	1

The radionuclides of interest for production of CR are listed in this table, and are modeled in this work. Characteristics of each radionuclide are given including half life, total abundance and abundance of emissions greater than the threshold for CR production. The CR abundance efficiencies are given for 1) water (refractive index 1.33, threshold 263 keV) and 2) mammalian tissues (refractive index 1.4, threshold 219 keV).

a s - seconds, m - minutes, d – days.

Notable in this table is the lack of CR producing emissions for Ac-225 which has been reported to be a strong light producer [6]. Our experiments with Ac-225 replicated this result so we thought to consider Ac-225's daughters which we expected to be in transient equilibrium with Ac-225 in our samples. Table 2 shows the CR capable abundances for Ac-225's daughter radionuclides along with their relative activities at transient equilibrium. These numbers suggest that Bi-213 and Pb-209 are the likely sources of the bulk of the detected CR.

**Table 2:** Ac-225 daughters abundance of emissions and those having energy greater than the CR threshold.

Ac-225 daughters	Half life^a^	% of Ac-225 activity at transient equilibrium	β^+^ (%)	β^−^ (%)			conversion electrons (%)			γ-rays (%)		
				total	1.33	1.4	total	1.33	1.4	total	1.33	1.4
Fr-221	4.9 m	100					6	0	0	12	0	0
At-217	32.3 ms	100					<0.1			<0.1		
Bi-213	45.59 m	100		98	65	71	5	5	5	27	27	27
Tl-209	2.20 m	2.2		100	81	85	29	4	4	282	198	198
Po-213	4.2 µs	97.8					<0.1			<0.1		
Pb-209	3.253 h	100.01		100	28	35						
Bi-209	stable											

The alpha-emitting radionuclide Ac-225 has been identified as a strong producer of CR light. Assuming their stable equilibrium with Ac-225, we list the relative activities of the daughters. In this table we also list the characteristics of the daughter radionuclides, their total abundance and their capabilities to produce CR in water and tissue.

a s - seconds, m - minutes, d - days.

We also note that In-114m (see Table 1) is a common long-lived impurity in samples of In-111. In-114m, in turn, decays to In-114. Because of In-114's short-half life its activity level in samples is in secular equilibrium with the In-114m within a few minutes and thus the two will have roughly equal activity levels. Samples of In-111 that are to be used clinically can have In-114m activity levels up to 0.15% [20] (and therefore an equal fraction of In-114) and this fraction will increase over time given In-111's faster rate of decay. In-114 has significant CR production potential from its highly abundant high energy β^−^ emission (see Table 1).

#### Modeling Cerenkov production per β of a given initial energy

The production of CR from a β particle is described by the Frank-Tamm formula [21] here integrated over a range of wavelengths.
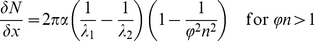
(1)

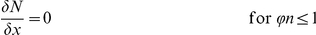



This formula gives the number of Cerenkov photons generated per unit path length having wavelengths within the interval from 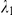 to 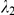 expressed in the desired length unit. Here 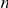 is the mean refractive index and 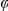 is the velocity of the β-particle divided by the speed of light in a vacuum and 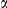 is the fine structure constant.

The β-particle velocity, 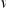, can be determined from its energy, 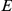, as follows:. 
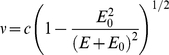
(2)where 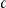 is the speed of light in a vacuum and 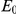 is the rest mass of the β-particle expressed in the same units as 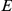.

For a given initial energy, we used Euler's method to integrate equation (1) over the full path length of the β-particle as it decreased in energy while transiting through a medium presumed to be of infinite spatial extent. During this integration, the rate of energy loss was interpolated from the ESTAR Stopping Power and Range Tables provided by the National Institute of Standards and Technology (NIST) [22]. The table used in our model had 250 logarithmically spaced points between 1 keV and 10 MeV. The energy step size for the Euler integration was 0.1 percent of the instantaneous β energy or 0.1 keV, whichever was larger. The total path lengths calculated, implicit in this process, were found to have a maximum error of 0.3% relative to the CSDA (continuous slowing down approximation reported by NIST) within the range of sampled energies. It should be noted that the full path length was calculated for testing purposes only. During routine use the integration for each β is terminated when its energy drops below the CR threshold.

#### Modeling Cerenkov production by β's and conversion electrons

In order to determine the average number of Cerenkov photons produced by β particles (or equivalently by conversion electrons) per disintegration for a given radionuclide, we weighted the above described integral by the relative probability of a β of a given energy being emitted by that radionuclide and then summed across all possible energies (i.e. a third integration of the original Frank-Tamm formula). The probability for each β energy was derived from the β spectra available from the Lund/LBNL Nuclear Data Search website [17]. The spectra were sampled at 1 keV intervals.

#### Modeling Cerenkov point spread function

In addition to the above described numerical models relating absolute number of Cerenkov photons to initial β energy, we developed a Monte-Carlo model to determine the Cerenkov point spread function (PSF) for a given radionuclide. In order to incorporate this spatial information, this model calculates the tortured path that each β particle makes as it generates Cerenkov photons and scatters off nuclei and electrons within the medium.

Our model of this transport process closely followed the work of Levin and Hoffman who modeled positron transport for the purpose of determining the positron-electron annihilation PSF for various radionuclides [23], [24]. In brief, we too made use of Bethe's calculations of Moliere's theory of multiple elastic scattering from the nucleus [25] and Ritson's model of the δ-ray energy distribution [26]. For details, please see the original manuscripts. Our model differed from Levin's in that instead of using the Bethe-Bloch formula to determine the collisional energy loss rate, we used the aforementioned ESTAR table. And, instead of using the Wu and Moskowski and Daniel models of β energy spectra, we again used the aforementioned tables from Lund/LBNL. As a check on the accuracy of our model, we calculated the positron PSF for some of the same radionuclides described by Levin and found the results to be comparable.

At each step of the β transport, we calculated the number of Cerenkov photons produced based on the length of the step and on the energy of the β at the start of the step (applying equation 1 as before). The location of the Cerenkov photons was distributed linearly along the path of the β for that step. Any δ-rays of sufficient energy were set to generate Cerenkov photons in the same manner as the β particles.

#### Modeling Cerenkov from secondary electrons excited by γ-rays and annihilation photons

Secondary electrons (i.e. photoelectrons) produce CR in a manner identical to that of β^−^ particles. However, the location of the Cerenkov production is generally far away from the originating radionuclide. This is because the γ-rays or annihilation photons will often travel a long distance before undergoing the photoelectric or Compton interaction that ultimately gives rise to a secondary electron. As such, the total amount of CR produced by secondary electrons will be geometry dependent. Larger volumes will tend to have a larger fraction of total Cerenkov signal produced by secondary electrons but this is contingent on annihilation photons and/or γ-rays of sufficient energy to produce secondary electrons capable of producing Cerenkov photons within the given medium.

To investigate and quantify this effect, we created two Monte Carlo model variants describing the transport of γ-ray and annihilation photons. The first consisted of a radionuclide point source within an infinite medium. This model was used to determine the PSF of CR due to secondary electrons. The second variant consisted of radionuclide evenly distributed within a cuboid-shaped medium. This model was specifically intended to mimic the conditions of our phantom studies (described below) that were designed to calibrate our luminescence scanner.

In both of these models, the initial directions of the simulated γ-rays emanating from the radionuclide source were randomly sampled so as to be uniformly distributed within the 4π solid angle about the source. Annihilation photon directions were similarly distributed but were created in pairs with members traveling in opposite directions. The distance traveled by each photon before interacting with the medium was randomly sampled from an exponential distribution, the log-slope of which was interpolated from a table of photon cross-sections, XCOM, available from the NIST website [27]. The table in our code used the standard grid available on the website but truncated to have energies between 1 keV and 10 MeV.

The total cross-section was used to determine the distance the photon traveled before interacting but the type of interaction, photoelectric, Compton or other, was randomly sampled reflecting the relative probabilities of each. When simulating a photoelectric interaction, all of the photon's energy was transferred to the secondary electron and the photon was terminated. For a Compton interaction, the Klein-Nishina formula was used to determine the scattering angle into which the photon was propagated, as well as the associated amount of energy transferred to the secondary electron. The CR associated with the secondary electron, if any, was determined from a lookup table calculated by our Cerenkov model based on the electron's initial energy (i.e. the path integral of equation 1). For the PSF model, all Cerenkov radiation was attributed to the site of the photoelectric or Compton interaction. All other types of interaction were assumed *not* to produce CR (e.g. pair-production was ignored).

### Experimental Cerenkov measurements

In order to test our models, five types of experiments were conducted (see Table 3). In one type of experiment, we acquired a luminescence spectrum. In a second type, we varied the refractive index of the medium (water) by adding 25% by weight of sodium chloride. In the third type, the volume of the medium was varied while maintaining a constant amount of radionuclide, thus achieving a range of surface to volume ratios and radionuclide concentrations. All measurements for these first three types of experiment were made using one of three simple acrylic boxes having 2 mm thick walls; one had a 3.4×3.4×3.4 cm interior, another was 5.4×5.4×5.4 cm and the third was 9.6×9.6×9.6 cm. Henceforth these will be referred to as the 3.4, 5.4 and 9.6 cm boxes respectively. All three were painted on all surfaces with flat black spray paint (Krylon Fusion). Tests on the boxes without radionuclide present demonstrated that they did not phosphoresce significantly following exposure to visible light.

**Table 3:** Radionuclides tested and the types of experiments conducted on each.

	Experiment Conducted (and Number)				
Radionuclide	Spectrum only (1)	Refractive index (2)	Volume change (3)	β PSF (4)	Secondary electron PSF (5)
F-18	X	X	X	X	X
Ga-68	X	X		X	
Zr-89	X	X	X		
In-111	X	X			
I-131	X	X			
Ac-225	X				

Experiments were used to validate the computation model presented. This table lists the experiment types, as well as the radionuclides employed to evaluate them.

The remaining two types of experiment were designed to measure the beta and secondary electron PSF's. The fourth experiment type, measuring the beta PSF, used a 5×5×3 cm solid acrylic block into which was cut a 0.11×3 cm by 1 cm deep slot on the 5×5 cm surface. The slot was filled with a mixture of radionuclide, India ink and surfactant. The India ink significantly reduced the CR emanating from the slot itself, leaving predominantly CR produced in the acrylic, which was taken to have a refractive index of 1.491 [28]. The surfactant allowed the slot to be filled without significant air pockets.

The fifth experiment type, which measured the secondary electron PSF, made use of a 10×10×5 cm solid acrylic block onto which a small drop of radionuclide was placed in the center of its 10×10 cm face.

All CR measurements were made on Caliper Life Science's IVIS 200 luminescence imager with the phantom placed in the center of a 13×13 cm field-of-view. The camera focus was set at 1.5 cm above the platform (i.e. the surface on which the box rested). The IVIS 200 uses a fixed focus lens and adjusts the focal point by adjusting the platform height relative to the camera. The 1.5 cm setting is the default focus point and we used this setting regardless of the height of fluid contained within the box. For the acrylic block phantom measurements, the camera was focused on the proximal surface of the block. All luminescence images were taken with an f-stop of *f*1 and a binning of 4 (i.e. 2×2 groups of pixels summed). Cosmic ray and background corrections were turned on. Total radioactivity of the radionuclide samples were measured with a Capintec Model CRC-127R dose calibrator (Capintec, Inc. Ramsey, NJ).

The IVIS 200 uses a cooled, back-thinned CCD (charge coupled device) detector. The signal measured from each pixel of this detector is roughly proportional to the number of photons impinging on the element during an image acquisition frame. The lens that focuses the light on the CCD includes an aperture which defines the solid angle of photon acceptance at a given focus point distance. The focus distance, along with the focal length of the lens, determines the surface area seen by each pixel. Thus the images acquired by the IVIS can be calibrated to photons per second per cm^2^ per steradian. For isotropic sources, the pixel values can be summed and multiplied by 4π steradians and by the area covered by the pixels in cm^2^ to arrive at the total photon flux in photons per second.

Although the direction in which Cerenkov photons propagate is dependent upon the direction of travel and energy of the charged particle, because the directions of the charged particles in our Cerenkov efficiency experiments are isotropic, so too on average are the Cerenkov photons. Note, this is not precisely true of the secondary electrons or their associated Cerenkov photons in our Cerenkov efficiency experiments. The initial direction of travel of secondary electrons relative to the parent photon direction is governed by the Klein-Nishina equation and is not strictly isotropic. However, as these electrons scatter producing CR along their path much of the directional bias is lost to the point where an isotropic assumption is acceptable. A similar reasoning applies to the β's and associated Cerenkov photons in our PSF measurements.

The photon attenuation of deionized water and 25 percent NaCl in water is negligible over the range of wavelengths of our spectral measurements (a max of 0.00439 cm^−1^ within the 550 to 670 nanometer range [29]). Thus, the total photon flux (within a range of wavelengths), after applying the corrections described below, is a direct estimate of the total CR production by the total radioactivity present. Thus the efficiency of the CR production can be simply calculated as the photon flux divided by the total radioactivity (e.g. photons per second per becquerel or equivalently, photons per disintegration). It should be noted that this means that the total photon flux measurement is by-in-large independent of the concentration of the radionuclide and that the pixel intensities varied predominantly with the surface area of the medium.

#### Spectral measurements

Spectral luminescence measurements were made using the six 20-nanometer band-pass filters available on the IVIS 200, centered at 560, 580, 600, 620, 640 and 660 nanometers. Immediately preceding these image acquisitions, one or more open (i.e. without filter) images were acquired, varying the frame duration until a reasonably low-noise image of the Cerenkov radiation was achieved. The filtered image frame durations were set to be 20 times that of the low-noise open image. The open measurements were used only to predict reasonable frame times for the filtered measurements. We did *not* model the open condition. Prior to the spectral acquisition and each of the open acquisitions, an associated reflected light image was acquired.

#### Cerenkov efficiency as a function of refractive index

Radionuclide, initially in a volume no greater than 1 mL, was thoroughly mixed with deionized water achieving a total volume of 30 (or 100 in some experiments) mL at room temperature. The refractive index of this medium was taken to be 1.333 [28] with a density of 0.998 g/cc at 20°C [30]. This solution was then transferred to the 3.4 (or the 5.4) cm box and a set of spectral measurements made with the IVIS imager.

The solution was then temporarily transferred to a container with a closeable top, to which was added 10 (or 33.3) grams of NaCl and shaken to dissolution, thus achieving a 25% by weight salt solution assumed to have a refractive index of 1.377 [18] and density of 1.281 g/cc [30]. The salt solution's volume was measured before returning to the box container and imaging was conducted as before.

#### Cerenkov efficiency as a function of volume

Radioactivity was initially mixed with 10 mL of deionized water and imaged in the 3.4 cm box placed in the center of the field of view. Without moving the box, 15 mL of deionized water was added bring the total to 25 mL. It was allowed to mix thoroughly and was reimaged. This sequence was then repeated but this time adding 25 mL (for a total of 50 mL). The whole volume was then transferred to the 9.6 cm box, also placed in the center of the field of view. Another 50 mL of deionized water was added and imaged. The sequence was repeated two more times adding 150 and 250 mL for a total of 250 and 500 mL in the container, respectively.

#### Region of interest measurements and profiles

All region of interest measurements were made using the Living Image software (Caliper LifeSciences, Inc., Hopkinton, MA) which comes standard with the IVIS 200. This software is designed to provide quantitatively accurate images in radiance units (photons/second/cm^2^/steradian) and includes adjustments accounting for platform height, lens aperture setting, field inhomogeneity, pixel binning and various sources of background. For our Cerenkov efficiency measurements, we placed a large region of interest over the homogeneous region within and well away from the edges of the box containing the deionized water or salt solution medium. The mean radiance within this region was then multiplied by the known surface area of the box opening (e.g. 92.16 cm^2^ for the 9.6 cm box) and by 4*π* steradians to arrive at the total Cerenkov photon flux in photons per second. Calculating the total flux in this manner, effectively corrects for light lost due to β particles exiting into the side walls of the container.

The PSF profiles were measured using custom code written in Matlab (The MathWorks, Inc., Natick, MA). For the Cerenkov profile due to β emissions, this entailed first rotating the image so that the slot was precisely aligned with the vertical axis of the image and then summing along the length of the slot to generate a profile extending perpendicular to the slot.

For the Cerenkov profile due to secondary electrons, the center of the drop (i.e. the radionuclide source) was identified within the image and the mean radiance surrounding that central point was plotted as a function of distance from that point.

#### Corrections - IVIS recalibration

**Figure 1:**
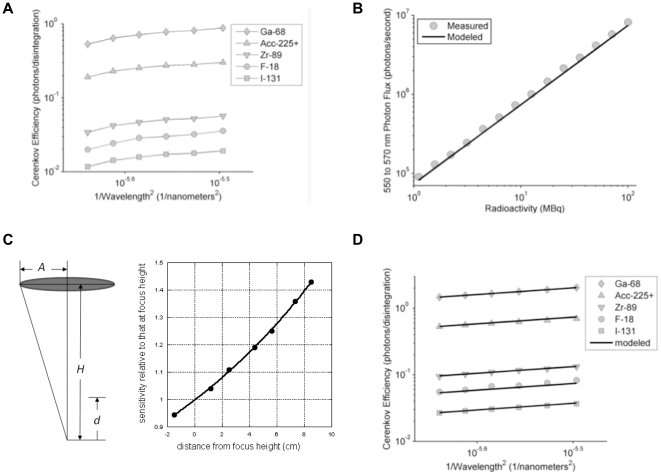
Evaluation and Correction of Luminescence Imaging System for CR. A) The CR efficiency measured as a function of one over the photon wavelength squared using calibrations provided by the manufacturer. These plots should be linear. B) Test of the linearity of the photon flux measurements. C) The diagram depicts the lens of the luminescence imager (gray ellipse) and defines the parameters used in expression (4). Plot on right shows the measured camera sensitivity as a function of the height of the imaged object (dark circles) along with a fit of expression (3) to determine the value for parameter H (which was otherwise difficult to measure directly). D) Same data as in (A) but now after calibrations based on our model and the spectral measurements for Ga-68. All measured spectral data are now very close to linear.

During the course of this work it quickly became apparent to us that neither the global absolute calibration nor the relative calibration of the individual 20 nm band-pass filters of our IVIS imager was consistent with our model's expectations. Specifically we noted that the Cerenkov spectra we were measuring did not have the characteristic one over wavelength squared shape we were expecting and yet the shape was consistent across radionuclides (Figure 1A). Examining the Cerenkov spectra published by others [5] we noted similarly consistent spectral curve shapes across radionuclides that were both different from the theoretical shape and different from that which we were measuring. We also noted, even after all corrections, that our Cerenkov measurements were consistently about half of that predicted by our models. Cleaning the lens and filters within the IVIS had a dramatic affect on the system's sensitivity but still failed to bring it in line with our expectations. For these reasons, we decided to recalibrate our IVIS based on a single spectral measurement of the Cerenkov light given off by Ga-68 in deionized water. This amounted to multiplying each of our filtered measurements by a slightly different factor ranging between 1.91 and 2.22. This same set of constants was used for all subsequent measurements (i.e. in effect we recalibrated the filters).

#### Corrections - Decay

All doses were calculated as the mean dose present during the interval over which the image was acquired. This was accomplished by applying a decay factor to the dose calibrator measurement. The decay factor, 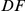, was calculated using the following well known formula:
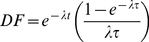
(3)where 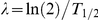, 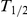 is the radionuclide half life, 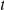 is the time between when the dose was measured and the start of the acquisition frame and 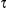 is the frame duration.

#### Corrections - Background

The standard image processing on the IVIS 200 includes a correction for the roughly uniform background typically encountered in luminescence imaging. However, in our measurements there was an additional source of background when imaging some radionuclides. This background is due to the direct detection of x-ray, γ-ray and/or annihilation photons by the luminescence detector. Because these high energy photons are not focused by the IVIS's lens system, this background too is fairly uniform. To correct for this background, we subtracted a constant from the luminescence image. The constant was determined by taking the mean value of a large region of interest placed a few cm away from the radionuclide source in each acquisition.

#### Corrections - Linearity

If all other things are held constant, the amount of Cerenkov radiation produced by a radionuclide is directly proportional to the amount of radioactivity present. Thus, given the well and accurately known half-life of F-18, for example, multiple measurements of Cerenkov light made as a radionuclide decays make for a good test of the linearity of a luminescence imaging system.

To test the linearity of our IVIS 200, we started with 3.5 mCi of F-18 diluted in 150 mL of deionized water, placed in the 5.4 cm box and imaged it repeatedly over 6.5 half-lives, 11.9 hours total. The frame duration (i.e. time the shutter was open) was held constant at 5 minutes for each measurement. Images were acquired every 54.885 minutes (i.e. 1/2 of F-18's 109.77 minute half-life) with the 560 nm (20 nm band pass) filter in place.

In Figure 1B is shown a scatter-plot of the radioactivity level versus the background corrected total photon flux rate measured in each image. The solid line shows the amount of Cerenkov light predicted by our model. Based on these results we determined that a linearity correction was not necessary.

#### Corrections - Source to camera distance

As a point source moves closer to the camera system, the solid angle limiting which photons have a chance of being detected by the camera, increases. Thus, closer objects appear brighter than more distant objects. For our phantom studies, the camera is detecting light from sources distributed throughout the depth of the liquid medium. Sources at shallower depths, therefore, are being detected with greater efficiency. This effect is described by the expression:
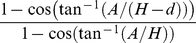
(4)where 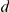 is the depth relative to a reference distance 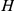 (e.g. the distance to the focus point) and 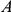 is the radius of the aperture at *f*1 which, for the IVIS 200, is 6.35 cm [31].

The value for 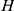 was determined by performing a nonlinear least-squares fit to a series of measurements of the total photon flux taken from a constant planar source positioned at various heights relative to the focus point (1.5 cm above the platform in all our measurements). This procedure found 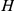 to be 51.2 cm. The parameter definitions, measurements and the fit are shown in Figure 1C.

In addition, because of the change in refractive index between the medium and the air above it, each plane at a given depth is magnified (a phenomenon well known to SCUBA diving enthusiasts, wherein objects under water appear to be closer than they really are). This magnification affect reduces the apparent radiance at a given depth in that the photons produced there appear to be generated over a larger surface area. Specifically, the magnification and thus the factor decrease in radiance is described by the following:
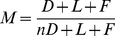
(5)where 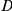 is the distance below the surface, 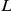 is the distance from the lens to the surface, 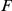 is the focal length of the lens and 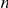 is the refractive index of the medium.

To arrive at a correction for measurements taken from fluids of differing depths, we averaged expression (4) divided by expression (5) over the entire depth of the fluid medium.

#### Corrections - Loss of Cerenkov at surfaces

The β-particles leaving the medium at its surfaces result in a loss of Cerenkov light production. This loss was estimated by the following:
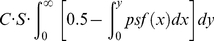
(6)where 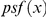 is the CR point spread function, 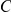 is the radionuclide radioactivity concentration, 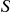 is the medium's surface area and 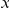 and 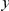 are both distances from the side of the container.

Because our measurements of total photon flux avoided losses at the sides of the box (by extrapolating the central homogeneous radiance to the edges), 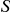 refers only to the area of the top and bottom surfaces. This expression assumes infinite extent for the dimensions parallel to the edge and does not consider the overlap at the edges and vertices of the containers, which will become significant as the container dimensions approach the full width half max of the PSF. For our containers, however, this does not incur a significant error given the PSF's considered here.

## Results

### Model Validation

#### Comparison of measured and modeled Cerenkov efficiencies

Following the recalibration of our IVIS 200 imager based upon our measurements of the Ga-68 CR spectrum, all spectral measurements of CR demonstrated the characteristic one over wavelength squared functional form (see Figure 1D). This was true for both our deionized water and salt solution measurements and for all radionuclides, including our Ac-225 and In-111 measurements. Moreover the magnitude of the predicted relative to the measured CR efficiencies, following the recalibration, were all within the error of our dose calibrator measurements.

**Figure 2:**
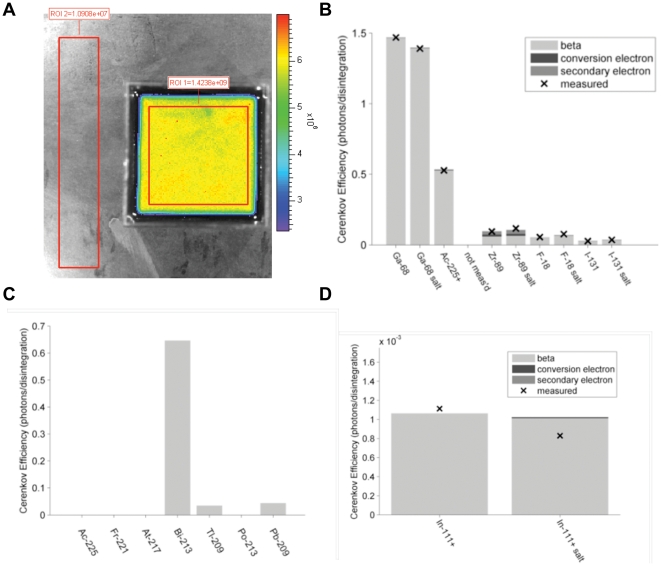
CR Efficiency Contributions From Three Sources; Modeled and Experimental Readings. A) The experimental setup is shown for a representative acquisition. The radionuclide was diluted in a defined medium and CR efficiency was measured and the background is subsequently subtracted. B) CR efficiency contributions from three sources, β-particles, conversion electrons and secondary electrons, as determined by our models along with comparisons to measured efficiencies. C) Contributions to CR production by Ac-225 and its daughters in deionized water as predicted by our model. D) Modeled and measured CR production efficiency for In-111 plus an assumed 0.05% impurity of In-114. All efficiencies shown are for the production of photons having wavelengths between 650 and 670 nanometers. The results are from experiments using deionized water and a 25% by weight sodium chloride and water solution (“salt”). Note - Ac-225+ denotes Ac-225 plus its daughters in transient equilibrium.

Figure 2A shows a representative image acquired during one of our Ga-68 box experiments. From this image one can appreciate the uniformity of the radiance emanating from the box having only a slight decline at the edge presumably due to escaping positron. Figure 2B shows a bar chart with bars breaking down the contributions for beta, conversion electron and secondary electron components for each radionuclide in water and in salt solution with X's showing measures made with the 660 +/− 10 nm band pass filter. Each of the measurements was made using a reasonably large volume of medium (∼100 mL except for I-131 which was made in ∼30 mL) and yet the CR contribution from secondary electrons in almost all cases was negligibly small. This was despite the high abundance of annihilation photons in Ga-68 and F-18. This can be understood by appreciating that Compton interactions in these mediums are far and away the dominant mechanism by which an annihilation photon (having a kinetic energy of 511 keV) gives rise to secondary electrons. Compton interactions allow a maximum transfer of energy to the secondary electron that is well below the energy of the photon; the so-called Compton edge. For a 511 keV photon, the maximum energy transfer in a Compton interaction is 340.7 keV. Most interactions transfer far less energy.

Zr-89 on the other hand has an appreciable contribution from secondary electrons. In this case, however, these are not primarily resultant from Zr-89's annihilation photons but rather from its 100% abundant 909 keV gamma which can transfer up to 709.6 keV in a Compton interaction. The related conversion electrons also contribute significantly to Zr-89's CR production efficiency.

The observant reader will also have noticed that Ga-68's CR efficiency in the salt solution medium is lower than that in deionized water, a trend that runs contrary to the usual increase with increasing refractive index (see below). The explanation for this can be found by noting that the salt solution also has a higher mass density and therefore a higher β attenuation cross-section and concomitant reduced β-particle path length. Increased density therefore tends to reduce CR production efficiency, but for radionuclides having relatively low energy β's, the increased refractive index overwhelms this reduction. For the high energy β's of Ga-68 however, the impact of refractive index is small and the density effect dominates.

#### Actinium-225 and Indium-111

As can be seen in Figure 2B, our model does an excellent job predicting the amount of CR produced by Ac-225 and its daughters when we assume that transient equilibrium has been reached. It should be noted that the dose calibrator setting we used (Capintec cal #775 with a 5× multiplier) to quantify the dose, makes a similar assumption. For the volume of medium used in this experiment, the contribution from secondary electrons (and from conversion electrons in general), were negligible, leaving β-particles from Ac-225's daughters as the predominant source. The CR contribution from Ac-225 itself is non-existent (see Figure 2C) and the vast majority of the CR signal is attributed to Bi-213.

Our model of In-111 in a 25% salt solution medium predicted a CR production efficiency of 2.57e-5 photons per disintegration within the 550 to 570 nanometer range. This is just 2.5% of the light within this range that was measured emanating from the In-111 sample in our experiment. In deionized water, our model predicted zero contribution from In-111. If, however, we assumed that In-114 was present as an impurity in the sample at a level of 0.05% (i.e. within the FDA allowed 0.15% for this unexpired sample), the measured and modeled came within reasonable agreement (see Figure 2D) especially considering that the background levels in these measurements were over 80% of the measured signal.

#### Comparison of measured and modeled Cerenkov from β, point-spread-functions

**Figure 3:**
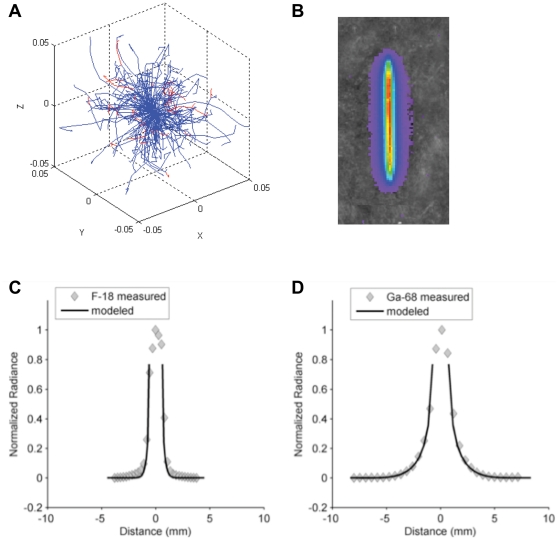
CR from β's Point Spread Functions. A) Simulated β^+^ tracks (blue) from an F-18 point source. Red tracks are from δ particles. B) A representative acquisition of the PSF experimental setup. This shows the channel in the acrylic block filled with a mixture of activity, surfactant and India ink. C) Integrated F-18 and D) Ga-68 measured radiance profiles shown as diamonds. Solid lines are modeled shapes with fitted amplitudes assuming β-particle source of CR.

Figure 3A shows a Monte Carlo simulation of the paths taken by 200 β^+^ particles emanating from a single point and having energies equivalent to those emanating from F-18. CR is produced all along these tracks until the β energy drops below the CR threshold.

Our experiments measuring the Cerenkov from β PSF used a roughly planar source of radioactivity and was integrated over the two axes parallel to this plane; the depth dimension integration being done implicitly by the camera resulting in an image (see figure 3B) and the other during the post processing of the images. As such, these experiments did not measure the PSF directly but rather they measured (approximately) the projection of this function onto the axis perpendicular to the plane. Therefore, we adjusted the output of our model, which calculates the distribution of Cerenkov light about a point source, projecting this light onto a single axis.

Figures 3C and D show the integrated PSF profiles from this type of experiment for F-18 and Ga-68, respectively. The profiles extend through and beyond the radionuclide containing slot (i.e. plane) in both directions and thus there are two independent measurements of the projected PSF with a gap (the width of the slot) in between. The India ink greatly diminished but did not eliminate the Cerenkov light emanating from the slot proper, hence the signal attributed to this region seen in the graphs. The solid lines are the modeled PSF projections (one a mirrored version of the other and separated by the known gap width) scaled somewhat arbitrarily so as to achieve a good fit to the measured data.

#### Comparison of measured and modeled Cerenkov from secondary electrons, point-spread-function

**Figure 4:**
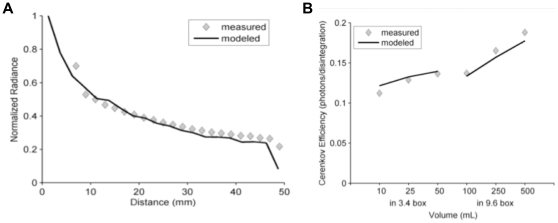
Volume Dependence of CR Production. A) Projected point spread function for F-18 drop placed on acrylic plastic. Measured radiance shown as diamonds. Solid line is modeled shape with fitted amplitude assuming secondary electron source of CR. B) CR efficiency of Zr-89 as a function of the dimensions of the deionized water medium. Measured values made using the 560 nanometer bandpass filter are shown as diamonds. Solid line is the modeled efficiency.

Our measurement of the Cerenkov from secondary electrons PSF, likewise, did not measure the PSF radial profile directly. Instead, in this measurement the camera first integrates the PSF over the depth dimension (i.e. that parallel to the direction in which the camera is pointing) and the resultant two-dimensional PSF is then projected onto a single radius during post processing. The output of the model was adjusted to mimic these projection operations and the result was scaled to fit the measured curve. The result is shown in Figure 4A.

As can be seen in this plot the tail of the PSF would actually extend beyond the dimensions of our block. A block large enough to measure the PSF in its entirety would not be able to fit in the light tight enclosure of our IVIS camera system.

#### Comparison of measured and modeled volume dependence

Our model of the loss of CR due to β's and conversion electrons near the exterior surfaces in our experiments suggest that this affect is negligibly small for the volumes we used. As noted previously though, CR production attributed to secondary electrons is expected to increase with increases in the overall size of the medium. Figure 4B shows this dependency for the one radionuclide that we looked at having a significant CR contribution from secondary electrons, Zr-89. The predictions closely match the measured efficiencies.

### Extrapolation of model results to other common radionuclides

#### Modeled Cerenkov production efficiencies as a function of refractive index

**Figure 5:**
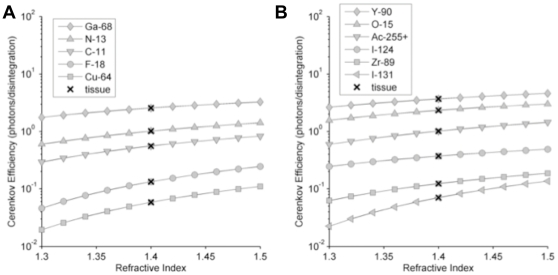
Modeled Cerenkov production efficiencies as a function of refractive index. Curves are the modeled efficiencies for β-particle produced CR as a function of refractive index assuming β cross-section properties and density of water. Efficiencies are in photons within the 550 to 570 nm range per disintegration. The X's used the β cross section properties of biological tissue. (A) and (B) list different radionuclides. Note - Ac-225+ denotes Ac-225 plus its daughters in transient equilibrium.

Having validated the accuracy of our models, we thought it would be beneficial to use the models to characterize a larger list of radionuclides so that investigators might use this information when selecting a CR producing radionuclide for a given purpose. Towards this end, we present in Figures 5A,B the CR production efficiencies for photons within the 550 to 570 nm range from β emissions predicted by our model and plotted as a function of refractive index for a variety or radionuclides. Other wavelength ranges can readily be calculated from this information by applying knowledge of the CR spectral shape. These curves assume a medium with β and γ cross-sections and density equal to that of water at 20°C, this in spite of the changing refractive index. While this is not entirely realistic, we felt the curves would be informative and reasonably accurate for water-like mediums such as biological tissue. To highlight this point we've included, on the same graph, points calculated for the cross-section [32], density and refractive index of tissue [19]. These efficiencies are also shown in Table 4 for the reader's convenience. The values shown do not include the CR production attributed to conversion electrons or to secondary electrons, which for the small animal geometries where this information is likely to be applied, are both expected to be small.

**Table 4:** CR from β Efficiencies.

Radionuclide	Efficiency	Radionuclide	Efficiency
C-11	0.5568	Zr-89	0.1230
N-13	1.0132	Y-90	3.7047
O-15	2.3301	I-124	0.3718
F-18	0.1328	I-131	0.0703
Cu-64	0.0583	Ac-225+	1.0143
Ga-68	2.5607		

The CR efficiencies for the radionuclides modeled in Figure 5A,B at the refractive index of tissue (1.4) are listed for convenience. Efficiencies are in photons within the 550 to 570 nm range per disintegration. Ac-225+ denotes Ac-225 plus its daughters in transient equilibrium.

As can be appreciated in these curves, CR production efficiency generally increases with increasing refractive index but the rate of this increase is radionuclide dependent. Generally speaking, radionuclides having higher energy β emissions will have a lower proportional increase in CR per unit increase in refractive index whereas radionuclides having β's closer to the CR threshold will have a greater proportional increase.

#### Modeled Cerenkov point spread functions

The Cerenkov from β PSF (prior to projection) is radially symmetric and therefore is described by its projection onto a single radius (i.e. integration over all angles). The resultant profile, it turns out, is reasonably well described by a sum of two exponentials. In order to arrive at robust values for the full-width at half-max (FWHM) and full-width at tenth-max (FWTM) values for this profile, we chose to fit our Monte-Carlo modeled data with a sum of two exponentials and calculate the metrics from the fitted curves using the modeled maximum value as the peak value. We present the results in Table 5 for our simulations of several commonly used radionuclides in biological tissue (i.e. refractive index 1.4 and tissue β attenuation).

**Table 5:** CR from β PSF width metrics.

Radionuclide	FWHM	FWTM	Radionuclide	FWHM	FWTM
C-11	0.712	1.824	Zr-89	0.712	1.664
N-13	0.816	2.330	Y-90	1.082	5.010
O-15	0.928	3.644	I-124	0.882	3.406
F-18	0.492	1.066	I-131	0.490	1.086
Cu-64	0.492	1.080	Ac-225+	0.790	2.194
Ga-68	0.928	3.996			

Here we list the PSF of the modeled radionuclides at the refractive index of tissue (1.4). FWHM and FWTM values are in mm. Ac-225+ denotes Ac-225 plus its daughters in transient equilibrium.

## Discussion

We have developed a set of models that accurately predict the CR production efficiency of various radionuclides through two mechanisms, directly from emitted β particles (and equivalently from conversion electrons) and from secondary electrons produced by the radionuclide's γ-rays or annihilation photons. The models allow both the refractive index and the photon cross-sections of the medium to be varied and thus should work for a variety of materials, including biological tissues. We've applied these models in two geometries (a point source in an infinite medium and a uniformly filled cuboid medium) and validated them experimentally. These models can be readily adapted to geometries of arbitrary shape and source distribution.

In addition, we have used these models to tabulate, for a number of commonly used medical radionuclides, the CR production efficiency and parameters describing the β particle and secondary electron Cerenkov point spread functions. This information can be used to evaluate which radionuclides are most suitable for a given application.

In 1969, HH Ross [33] modeled CR based counting of β emissions as an alternative to scintillation counting for radionuclide calibration purposes. Our work builds on Ross' with improvements in accuracy and extensions specifically suited to imaging applications.

While our manuscript was under initial review, a paper by Mitchell et. al. that described modeling of Cerenkov production was published [34]. Our work differed from theirs in that their models utilized Monte Carlo techniques at an earlier stage and they do not attempt to quantitatively confirm many of their results. Although their model was based on entirely different computer code, the Cerenkov efficiency results they reported are virtually identical to the values we calculate with our model.

Since the radioactivity level of many radionuclides can be determined with great accuracy, the Cerenkov efficiency information allows for a simple means of calibrating imaging systems capable of measuring low levels of light. Pure positron emitters (such as F-18 or Ga-68) in water will have little volume dependency and can be calibrated accurately in a dose calibrator. Ga-68 in particular is insensitive to small changes in refractive index in the vicinity of 1.33 and thus measurements from it are robust to temperature fluctuations and other factors affecting the refractive index of the medium. Using a simple setup, such as one of the boxes we described, the measured light in photons per second corresponds directly to the total dose of radionuclide. The corrections for background, source to camera distance and surface loss were all very small; as was the photoelectron contribution. Thus a simple multiple integration of the Frank-Tamm formula provides a robust and near direct estimate of the true photon flux.

We investigated the mechanism of the light production for two radionuclides, Ac-225 and In-111, for which the Cerenkov mechanism was called into question. Our analysis suggests that Ac-225 per se does *not* generate Cerenkov light, but that one of its β emitting daughters, Bi-213, is responsible for the bulk of the Cerenkov signal with significant contributions from Tl-209 and Pb-209. For In-111 we found that although it is theoretically capable of producing CR, the amount of light produced is extremely small and significantly smaller than that which was measured. We show evidence that the amount of CR produced is consistent with an In-114 impurity as its source.

As mentioned in the Introduction, our primary goal in developing these models is to determine the amount of CR produced by radionuclides placed within biological tissues. For this purpose, accurate knowledge of the refractive index of the tissue is necessary. However, there is a fair amount of uncertainty in the literature regarding the refractive indices of tissues [35] and even small differences can have a large impact on the amount of CR produced. There is also likely to be variation from one organ to another within the animal and certainly the refractive index will be very different for structures such as the urinary bladder. Radionuclides having higher energy β's are less sensitive to these variations in refractive index and therefore may be more desirable although at the cost of a reduction in resolution. In another context, the application of a controlled electron energy source may prove to be an accurate method of assaying the refractive index of a given tissue.

In future work we will be applying our models of CR production in order to validate and compare bioluminescence tomography reconstruction algorithms.
